# Vibrational Spectroscopy for Imaging Single Microbial Cells in Complex Biological Samples

**DOI:** 10.3389/fmicb.2017.00675

**Published:** 2017-04-13

**Authors:** Jesse P. Harrison, David Berry

**Affiliations:** Division of Microbial Ecology, Department of Microbiology and Ecosystem Science, Research Network “Chemistry Meets Microbiology”, University of ViennaVienna, Austria

**Keywords:** imaging, isotope labeling, single-cell analysis, vibrational spectroscopy

## Abstract

Vibrational spectroscopy is increasingly used for the rapid and non-destructive imaging of environmental and medical samples. Both Raman and Fourier-transform infrared (FT-IR) imaging have been applied to obtain detailed information on the chemical composition of biological materials, ranging from single microbial cells to tissues. Due to its compatibility with methods such as stable isotope labeling for the monitoring of cellular activities, vibrational spectroscopy also holds considerable power as a tool in microbial ecology. Chemical imaging of undisturbed biological systems (such as live cells in their native habitats) presents unique challenges due to the physical and chemical complexity of the samples, potential for spectral interference, and frequent need for real-time measurements. This Mini Review provides a critical synthesis of recent applications of Raman and FT-IR spectroscopy for characterizing complex biological samples, with a focus on developments in single-cell imaging. We also discuss how new spectroscopic methods could be used to overcome current limitations of single-cell analyses. Given the inherent complementarity of Raman and FT-IR spectroscopic methods, we discuss how combining these approaches could enable us to obtain new insights into biological activities either *in situ* or under conditions that simulate selected properties of the natural environment.

## Introduction

Natural habitats are often physically and chemically complex, which has far-reaching consequences for the spatial distribution of microbial taxa and the processes they mediate (Resat et al., 2012; Vos et al., 2013; Pande et al., 2016; Ratzke and Gore, 2016). Because controlled laboratory experiments rarely capture the heterogeneity present within natural environments, our knowledge of microbial activities is often based on indirect observation. To address this source of uncertainty, there is a need for methods that facilitate the *in situ* profiling of microorganisms and their activities in complex environments. A full understanding of these topics also requires an ability to study these processes at the level of single cells (Fike et al., 2008; Resat et al., 2012; Roose et al., 2016). Due to its ability to rapidly and non-destructively probe the physiology and activities of microorganisms, vibrational (Raman and FT-IR) microspectroscopy (a combination of microscopy and spectroscopy) shows considerable promise in this respect (Escoriza et al., 2006; Wagner, 2009; Lu et al., 2011). In particular, Raman and infrared imaging have emerged as useful methods for the spatially resolved analysis of biological samples. In this Mini Review, we highlight recent studies that have used these techniques to image single microbial cells within spatially and chemically complex environments. These include pure cultures incubated in contact with physical substrata, multi-species assemblages within their native habitats, as well as other challenging sample types. State-of-the-art approaches for spectral imaging are critically evaluated in order to identify guidelines for future applications of single-cell analyses in microbial ecology.

## Raman Imaging

While several types of Raman spectroscopic instrumentation and analytical approaches have been developed, each of these relies on measuring the scattering of monochromatic light as it interacts with a sample. Most photons are elastically scattered and possess the same energy as the incident light beam (also termed Rayleigh scattering). However, a small fraction is inelastically scattered, involving a decrease or an increase in energy compared with the excitation wavelength (Stokes and anti-Stokes Raman scattering, respectively). By providing information on vibrational and other low-frequency transitions in a molecule, both types of inelastically scattered light can be used to determine and differentiate between the chemical composition of solids, liquids and gases. For a more detailed introduction to this technique (as well as infrared spectroscopy), the reader is referred to Skoog et al. (2007) and Lu et al. (2011). Recent advances in the design of high-speed Raman imaging instrumentation have been summarized by Ando et al. (2016). Moreover, developments concerning techniques including surface- and tip-enhanced Raman scattering (SERS and TERS), as well as resonance Raman and coherent anti-Stokes Raman spectroscopy (CARS), are discussed in several reviews (Opilik et al., 2013; Camp and Cicerone, 2015; Cicerone, 2016; Kano et al., 2016).

Two features that make Raman microspectroscopy an ideal technique for single-cell analyses include its direct compatibility with aqueous samples (due to water exhibiting only weak Raman scattering) and its high spatial resolution (Skoog et al., 2007). While a resolution of ∼1 μm is possible using conventional Raman instrumentation, measurements at the nanometer scale are achievable by TERS (Mariani et al., 2010; Opilik et al., 2013; Rusciano et al., 2014). Raman measurements are also well-suited for analyzing motile cells using optical tweezers, as well as monitoring microbial activities by stable isotope probing (SIP) (Chan et al., 2004; Wagner, 2009; Huang et al., 2010; Berry et al., 2015; Wang et al., 2016). Although the real-time Raman imaging of microorganisms remains non-trivial due to issues including background autofluorescence (Polisetti et al., 2016) and weak signal intensities (partly due to a need for low laser excitation power to avoid photodamage), significant progress in this field has already been made. For example, Li et al. (2012) used resonance Raman imaging combined with ^13^C labeling to identify cells that fixed carbon dioxide in culture and in field-collected seawater samples. By reducing spectral acquisition times to milliseconds, resonance Raman spectroscopy – a method in which the excitation wavelength matches the electronic transition of a selected molecule – was key to enabling the rapid imaging of these samples. It is also possible to visualize selected strains and their locations within habitats including human endothelial cells (Große et al., 2015), macrophages (Silge et al., 2015) and other environments, even when the taxa of interest are present at low abundances (Kalasinsky et al., 2007). Through combining imaging of *Staphylococcus aureus* cells with a multivariate classification model [based on principal component analysis (PCA) and linear discriminant analysis (LDA)], Große et al. (2015) were further able to detect small differences in the spectral profiles that allowed the authors to discern between intra- and extracellular cells, due to shifts in the physiological state of the bacteria that occur upon host invasion.

In addition, resonance Raman and SERS have been used to directly image rhizosphere bacteria (*Pantoea* sp. YR343) on *Arabidopsis thaliana* root surfaces (Polisetti et al., 2016). This is of interest because Raman-based investigations of plant–microbial interactions are often challenging or impossible due to the strong autofluorescence originating from plant materials. In the study by Polisetti et al. (2016), background interference from the roots was reduced by aging them for 5–15 days. Similar to Große et al. (2015), PCA was used to discriminate bacterial spectra from spectra of other materials. Moreover, using SERS allowed the authors to circumvent the need for a photo-bleaching step which is often employed for the analysis of pigmented cells using conventional Raman instrumentation, but which can result in the degradation of cell components and metabolites that are of importance to understanding bacterially mediated processes in the rhizosphere (Polisetti et al., 2016). Taken together, the studies highlighted above illustrate how advanced Raman imaging techniques and multivariate analyses can be used to generate new insights into the distribution and activities of microorganisms within diverse environments, including systems which have previously been difficult to visualize and where the ability to differentiate between cells and other materials is dependent on detecting minor differences in spectral features. By removing the need for sample treatment steps that are likely to introduce analytical biases, such as sample photo-bleaching prior to the collection of Raman spectra (Polisetti et al., 2016), these techniques can also provide increasingly accurate information on metabolic processes occurring at multiple levels of biological organization (from individual cells to communities).

While a limited number of studies have been published on Raman imaging of microbial strains or uncultured cells within their native environments, new instrumentation is likely to lead to an expansion of this field by enabling reduced spectral acquisition times without a loss of signal intensity (Opilik et al., 2013; Ando et al., 2016; Kano et al., 2016). In addition, combining this approach with well-established methods in microbial ecology (including fluorescence *in situ* hybridization) (Wang et al., 2016) as well as newer techniques such as bioorthogonal chemical imaging (Berry et al., 2015; Wei et al., 2016) and Raman microfluidics (Chrimes et al., 2013) are likely to find increasing use in the analysis of microbiological samples. Several sample types which have not yet been subjected to Raman imaging have already been characterized using single-point measurements, and therefore represent promising targets for future research. For example, while the Raman-based detection of meningitis-causing pathogens in human cerebrospinal fluid has been achieved (Harz et al., 2009), spatially resolved imaging of such samples could facilitate the development of improved diagnostic tests. One imaging modality that is particularly promising from a microbiological perspective, but which is yet to find widespread use in the field of microbial ecology, is CARS (Krafft et al., 2009; Camp and Cicerone, 2015; Cicerone, 2016). This technique can enable the acquisition of Raman spectra at a rate that is approximately 100 times faster than conventional Raman analyses, making it highly suitable for the real-time imaging of biological samples (Cicerone, 2016). CARS has already been used for the rapid profiling of microorganisms at the subcellular level (Okuno et al., 2010; Yue and Cheng, 2016), and a single study has also employed it to image bacteria within complex matrices including milk and urine (Hong et al., 2016). Another technique which has found surprisingly limited use in the field of microbial ecology is TERS (Mariani et al., 2010; Opilik et al., 2013; Rusciano et al., 2014). However, since this method enables Raman measurements at sub-micron spatial scales, it could be used to analyze microorganisms that are under the conventional size detection limit of ∼1 μm, as well as viral particles present within diverse environmental matrices. Indeed, TERS has already been used for the analysis and classification of viral strains (Hermann et al., 2011; Olschewski et al., 2015).

## FT-IR Imaging

While Raman spectroscopy relies on irradiating a sample with a monochromatic laser beam, Fourier-transform infrared (FT-IR) spectroscopy is based on measuring the absorption of polychromatic infrared light. The functional groups in a given molecule are identified according to their vibrational modes at different IR frequencies (for detailed information, see Skoog et al., 2007). Raman analyses depend on a shift in the polarizability of a molecule, whereas FT-IR measurements depend on changes in the dipole moment. Indeed, Raman-active vibrational modes often exhibit weak IR signals and vice versa (with symmetric and asymmetric moieties producing strong Raman and IR spectral bands, respectively), and the two methods provide complementary information on the molecular composition of microbial cells (Lu et al., 2011; Ojeda and Dittrich, 2012; Tang et al., 2013; Wang et al., 2016). Infrared imaging could, therefore, provide insights into microbial physiology in samples that are difficult to analyze using Raman spectroscopy alone. Indeed, high-speed imaging of large (centimeter-scale) sample areas can be achieved using a focal plane array (FPA) detector that enables the simultaneous acquisition of tens of thousands of IR spectra (Dorling and Baker, 2013). Studies employing FPA-based FT-IR analysis are common in biomedical science and have, for example, involved chemical imaging of tissues (Kastyak-Ibrahim et al., 2012; Miller et al., 2013) and cancer cells (Kuimova et al., 2009). Chemical mapping by reflectance FT-IR microspectroscopy has also been used to characterize bacteria on opaque steel surfaces, without a need for destructive sampling (Ojeda et al., 2009). In comparison with Raman analyses, however, few studies have used FT-IR microspectroscopy to investigate single microbial cells within their native environments, potentially due to the coarse spatial resolution (∼10 μm) of conventional FT-IR measurements and water being a strong absorber of IR radiation. Even so, several ways to overcome these challenges have been developed. For example, synchrotron radiation sources have enabled FT-IR measurements at the micron scale (Nasse et al., 2011; Jamme et al., 2013; Saulou et al., 2013) and combining this approach with microfluidics can reduce background interference from water by making it possible to culture cells within a thin layer of fluid (Holman et al., 2009; Loutherback et al., 2015, 2016; Birarda et al., 2016).

While synchrotron-FT-IR analyses require dedicated facilities, advances in the development of high-magnification optics have made it possible to perform FPA-based infrared imaging at a spatial resolution comparable with Raman instruments, even without access to a synchrotron beamline (Findlay et al., 2015). Analyses of cells in aqueous suspensions are additionally possible using attenuated total reflectance (ATR)-FT-IR imaging (Kuimova et al., 2009). Where required, techniques for nano-scale infrared imaging have been developed (Reddy et al., 2013; Centrone, 2015; Amenabar et al., 2017) and even relatively thick aqueous samples can be analyzed by quantum cascade laser-based IR microspectroscopy (Haase et al., 2016). Crucially for the *in situ* analysis of microbial activities, there is evidence that FT-IR spectroscopy is compatible with SIP and can be used to track the cellular uptake of stable-isotope-labeled carbon (^13^C) and nitrogen (^15^N) compounds (Muhamadali et al., 2015). FT-IR microspectropy can detect differences in the spectra of water and heavy water (D_2_O), due to absorbance peaks corresponding to O–H and O–D bending modes occurring at different wavenumber regions (Miller et al., 2013). While we are unaware of studies that have combined D_2_O labeling with FT-IR spectroscopy to monitor the activities of individual microbial cells, this has recently been achieved using Raman spectroscopy (Berry et al., 2015), and it is likely that both methods can be used to identify actively metabolizing cells within their native habitats.

Further to the studies discussed above, Muhamadali et al. (2016) evaluated the applicability of three vibrational spectroscopy techniques (FT-IR, conventional Raman and SERS) for differentiating between several clinically relevant taxa including *Escherichia coli, Pseudomonas* spp., *Bacillus* spp. and *Enterococcus faecium*. Of these techniques, infrared spectroscopy was found to provide the most consistent results for the entire sample set (in terms of spectral quality and reproducibility), which led the authors to suggest that FT-IR analyses could be particularly useful for characterizing mixed cultures (also see Wenning et al., 2005). Indeed, FT-IR microspectroscopy has already been used to quantify compare the abundances of bacteria and archaea within subsurface aquifer samples, based on domain-specific CH_3_:CH_2_ absorbance ratios (Igisu et al., 2012). In comparison with Raman spectroscopy, there is evidence to suggest that FT-IR analyses can additionally give a higher degree of confidence when there is a need to discriminate between strains belonging to the same species (69 and 89% strain-level prediction accuracies for Raman and FT-IR, respectively, based on chemometric analysis; AlMasoud et al., 2016). Given these results, we anticipate infrared imaging to become an increasingly common technique in the field of microbial ecology, particularly when there is a need for quantitatively analyzing multi-species assemblages and/or in-depth physiological profiling of selected isolates.

## Recommendations and Outlook

The spectroscopic imaging of microbial cells in physically and chemically complex samples involves diverse analytical challenges. While addressing these will often require sample-specific optimization steps (such as identifying an appropriate laser wavelength; Edwards et al., 2003; Chan et al., 2004; Jorge Villar et al., 2005), many of them could be overcome by carefully selecting between Raman- and FT-IR-based measurements or a combination of both. Based on the case studies discussed in this Mini Review, it is possible to identify several general guidelines for achieving this (**Table 1**). The suggestions provided in **Table 1** additionally highlight the promising role that live-cell FT-IR imaging could play in environmental microbiological research, further to Raman measurements which have traditionally been more common in this field. The future development of vibrational spectroscopy instrumentation and analytical methods may serve to further enhance the cross-compatibility of Raman and FT-IR techniques (e.g., via improved access to advanced Raman imaging equipment and validation of new protocols for FT-IR-SIP).

**Table 1:** Experimental goals associated with the Raman and FT-IR imaging of single microbial cells in complex biological samples.

Goal	Recommended technique	Notes	Reference
Analysis of motile cells and/or cell sorting	Raman microspectroscopy	Optical tweezers can be used to trap or move individual cells	Huang et al., 2010; Berry et al., 2015
Detection of cells on autofluorescent and opaque surfaces	Both	Autofluorescence does not interfere with IR measurements; Raman measurements possible using resonance Raman, SERS, sample photobleaching or aging	Ojeda et al., 2009; Polisetti et al., 2016
Addressing other sources of background interference	Both	Water is a strong IR absorber; using microfluidics or an ATR accessory can reduce signal interference	Kuimova et al., 2009; Birarda et al., 2016; Loutherback et al., 2016
Stable isotope probing	Both	Approaches currently better-established for Raman analyses	Wang et al., 2016
Imaging of large (cm-scale) surface areas	FT-IR microspectroscopy	FPA detectors readily available for FT-IR instruments; Raman instrumentation also available, but not as widely accessible	Kuimova et al., 2009; Kastyak-Ibrahim et al., 2012; Miller et al., 2013; Ando et al., 2016
Localization of cells in 3D space	Raman microspectroscopy	Imaging of *z*-stacks possible using confocal Raman measurements	Große et al., 2015; Silge et al., 2015
High-resolution (including subcellular) measurements	Both	Raman analyses (e.g., TERS) better-established; also possible using FT-IR but requires specialist equipment or access to synchrotron beamline	Mariani et al., 2010; Opilik et al., 2013; Saulou et al., 2013; Rusciano et al., 2014; Findlay et al., 2015
Label-free discrimination between individual strains or taxa	FT-IR microspectroscopy	FT-IR analyses can outperform Raman spectroscopy in terms of spectral quality and reproducibility	AlMasoud et al., 2016; Muhamadali et al., 2016

*Recommendations for analytical techniques are provided for meeting each goal.*

**FIGURE 1:**
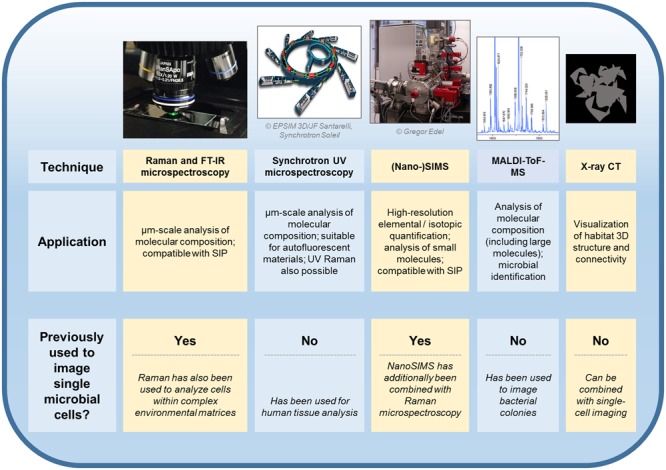
**Techniques which have or could be utilized for the *in situ* imaging of single microbial cells within physically and chemically complex environments.** Previously demonstrated applications of each approach are discussed in the main text. FT-IR, Fourier-transform infrared; SIP, stable isotope probing; UV, ultraviolet; (Nano-)SIMS, nanoscale secondary ion mass spectrometry; MALDI-ToF-MS, matrix-assisted laser desorption/ionization mass spectrometry time-of-flight mass spectrometry; CT, computed tomography.

Additionally to considering the benefits and pitfalls inherent to Raman vs. FT-IR measurements, experiments focusing on the imaging of single cells in complex habitats can be expected to profit from combining these techniques with other analytical approaches (**Figure 1**). Synchrotron-FT-IR microspectroscopy has been paired with synchrotron ultraviolet microspectroscopy and time-of-flight-secondary ion mass spectrometry (ToF-SIMS) for the analysis of human liver tissue, with each technique yielding unique information on the chemical composition of the sample (Petit et al., 2010). Raman microspectroscopy has been combined with nanoscale secondary ion mass spectrometry (NanoSIMS) to quantify the bacterial uptake of deuterium during heavy water labeling experiments (Berry et al., 2015). Moreover, matrix-assisted laser desorption/ionization time-of-flight mass spectrometry (MALDI-ToF-MS) is compatible with microbiological analyses and Raman imaging (Bocklitz et al., 2013; Pande et al., 2016; Stasulli and Shank, 2016). Although it has not yet been applied for the imaging of cells within complex environments such as soils, MALDI-ToF-MS been used to characterize individual bacterial colonies (Pande et al., 2016; Stasulli and Shank, 2016). Promisingly, the technique can be used for strain identification (Singhal et al., 2015) and a method for single-cell MALDI analyses has also been developed (Xiong et al., 2016). Vibrational spectroscopic imaging of microbial cells could be further combined with techniques that provide information on the 3D structure of the surrounding environment. X-ray computed tomography, for example, has been used to visualize roots within undisturbed soil (Mooney et al., 2012). The technique has also been used to produce micron-scale 3D representations of soil pore space (Nunan et al., 2006).

One of the most important challenges involved in the spectral imaging of microorganisms within their native habitats, regardless of the techniques involved, concerns the ability to successfully discriminate between cells and other materials. Additionally, an ability to discern between diverse taxa is required to understand the distribution and activities of microbial cells at the community level. To facilitate research into these topics, we strongly recommend that databases including relevant reference spectra are made available as part of future publications. We also note that using Raman and/or FT-IR spectroscopy alone for the reliable identification of microbial taxa often remains challenging (see FT-IR imaging), and that result using these methods may need to be verified using additional methods. For example, Raman-activated cell sorting has recently been combined with single-cell genomics to identify members of a novel cyanobacterial order within seawater samples (Song et al., 2017). Ultimately, the approaches discussed in this Mini Review could enable us to significantly improve our knowledge of microbial community assembly and the contribution of interspecies interactions to key ecosystem processes, including the cycling of carbon within soils, sediments and other spatially structured habitats.

## Author Contributions

All authors listed, have made substantial, direct and intellectual contribution to the work, and approved it for publication.

## Conflict of Interest Statement

The authors declare that the research was conducted in the absence of any commercial or financial relationships that could be construed as a potential conflict of interest.
